# The Oral Delivery System of Modified GLP-1 by Probiotics for T2DM

**DOI:** 10.3390/pharmaceutics15041202

**Published:** 2023-04-10

**Authors:** Qing Wang, Haixin Guo, Wenwei Mao, Xiuping Qian, Yangang Liu

**Affiliations:** 1 School of Pharmacy, Shanghai Jiao Tong University, Shanghai 200240, China; wangqing20@sjtu.edu.cn (Q.W.); wwmao@sjtu.edu.cn (W.M.); 2 Shanghai TriApex Biotechnology Co., Ltd., Shanghai 201315, China; hxguo@triapexbio.com

**Keywords:** type 2 diabetes mellitus, GLP-1, *Lactobacillus plantarum* WCFS1, oral delivery

## Abstract

The glucagon-like peptide-1 (GLP-1) is a peptide with incretin activity and plays an important role in glycemic control as well as the improvement of insulin resistance in type 2 diabetes mellitus (T2DM). However, the short half-life of the native GLP-1 in circulation poses difficulties for clinical practice. To improve the proteolytic stability and delivery properties of GLP-1, a protease-resistant modified GLP-1 (mGLP-1) was constructed with added arginine to ensure the structural integrity of the released mGLP-1 in vivo. The model probiotic *Lactobacillus plantarum* WCFS1 was chosen as the oral delivery vehicle with controllable endogenous genetic tools driven for mGLP-1 secretory constitutive expression. The feasibility of our design was explored in db/db mice which showed an improvement in diabetic symptoms related to decreased pancreatic glucagon, elevated pancreatic β-cell proportion, and increased insulin sensitivity. In conclusion, this study provides a novel strategy for the oral delivery of mGLP-1 and further probiotic transformation.

## 1. Introduction

The International Diabetes Federation (IDF) estimated the global population with diabetes mellitus (DM) to be 537 million in 2021 and projected it to be 643 million by 2030 [1]. Type 2 diabetes mellitus (T2DM) is the most common type of diabetes, which is characterized by a combination of interrelated metabolic disorders, including continuous hyperglycemia, peripheral insulin resistance, and dysfunction, loss, or dedifferentiation of pancreatic β cells [2,3]. The progression of T2DM is related to glycemic control since substantial functional β cell mass can be restored under good glycemic control or remission [4,5]. Currently, the glucagon-like peptide-1 (GLP-1), an endogenous intestinal hormone produced by L-cells of the distal ileum and colon, and its analogues have become one of the promising therapeutic drugs for the treatment of T2DM [6]. The peptide acts through GLP-1 receptors expressed in numerous tissues, such as the pancreas, kidney, stomach, and intestine, and has various antidiabetic effects including increasing insulin secretion, suppressing glucagon secretion, inhibiting gastric motility, and promoting satiety [7,8].

However, the half-life of the native form of GLP-1 in circulation is only 1–2 min due to the rapid cleavage and inactivation by the dipeptidyl peptidase-4 (DPP-4) [9]. Two strategies have been employed to overcome this limitation: DPP-4 inhibitors and long-circulating GLP-1 receptor agonists such as exenatide and semaglutide [7,9]. Moreover, the tirzepatide, a novel dual GLP-1 and glucose-dependent insulinotropic polypeptide (GIP) analogue, which performed well in phase III clinical trial, has been approved by the US Food and Drug Administration (FDA) and European Medicines Agency (EMA) for management of T2DM [5,10,11]. Nevertheless, the main route of administration of the GLP-1 receptor agonists that have been approved or used in clinical treatment is subcutaneous injection [7,10,12,13,14], which may cause subcutaneous lipoatrophy and poor patient compliance compared with oral administration [15,16,17]. Although semaglutide is now available for oral administration with sodium N-(8-[2-hydroxybenzoyl] amino) caprylate (SNAC) as an absorption enhancer, there is still some room for improvement due to its demanding administration requirements and higher cost caused by increased doses [18]. A more convenient and affordable method of delivery that could improve patient compliance and simulate the physiological release of GLP-1 is still urgently required in clinical practice.

Lactic acid bacteria (LAB), a kind of Gram-positive bacteria, is a normal flora of the gut microbiota of human hosts [19]. Some LAB species are officially recognized as “Generally Recognized as Safe” (GRAS) by the FDA [20]. Research indicates that LAB strains could exert probiotic effects such as maintenance of host gastrointestinal homeostasis [21], anti-tumor [22], immunity enhancement [23,24], blood lipids regulation [25], and blood glucose regulation [26]. Furthermore, some LAB strains could pass through the human gastrointestinal tract successfully and colonize the surface of intestinal epithelial cells in situ, protecting the mucosal-targeted delivery of the therapeutic drugs from gastrointestinal digestion and enzymatic degradation [27,28]. These intrinsic advantages of the LAB strains present an attractive alternative to the use of other mucosal delivery systems with potential problems caused by vehicles such as liposomes and micro-particles [29,30,31]. LAB strains, therefore, have gradually been recognized as one of the promising delivery vehicles for a range of compounds, such as antigens [32], peptides [33], enzymes [34], immune modulators [35], and gene therapy agents [36]. Notably, the first phase I clinical trial conducted in 2006 demonstrated that the application of genetically modified *Lactococcus lactis* was an effective method for the interleukin-10 topical delivery that circumvented systemic side effects and allowed long-term treatment of chronic diseases [35]. In 2021, the positive interim data from a Phase Ib/IIa Study of AG019 ActoBiotics™, another novel transgenic *Lactococcus lactis* product for type 1 diabetes mellitus (T1DM), was announced by Precigen ActoBio (Clinical Trial Identifier: NCT03751007).

*Lactobacillus plantarum* (*L. plantarum*) WCFS1 (isolated from NCIMB 8826), one of the model strains of LAB, has gradually become a promising expression and mucosal delivery vehicle due to the in-depth research and deepening understanding of it in recent years. The whole genome of *L. plantarum* WCFS1 was first sequenced in 2001 and updated in 2011, identifying the first genome of the *Lactobacillus* species [37,38]. To facilitate the efficient expression of the heterologous proteins, a set of controllable genetic tools have been developed for *L. plantarum* WCFS1. Modular plasmid expression systems, for example, consisting of replaceable elements such as the promoter, the cognate regulatory system, the target gene, and the antibiotic resistance gene have been developed. Among them, the pSIP-plasmid allows inducible expression using the regulatory machinery naturally involved in LAB bacteriocin production and has the potential for extended applications through module switching [39,40]. The consensus promoter sequence of *L. plantarum* WCFS1 was derived by aligning its rRNA promoters, and a synthetic promoter library (SPL) for *L. plantarum* WCFS1 was also established and evaluated subsequently in 2005 [41]. Given the challenges of inefficient secretion of heterologous signal peptides [34,42], a genome-wide homologous signal peptides (SPs) library of *L. plantarum* WCFS1 was also constructed in 2009 [43]. In addition, some surface modifications of *L. plantarum* WCFS1 were attempted to ensure the storage stability and the in situ mucosal delivery reliability of heterologous proteins [44]. Based on the above, and the fact that *L. plantarum* WCFS1 could colonize the colon and cecum of healthy volunteers and survive for 3–7 days [45,46], there have already been several inspiring examples of heterologous protein deliveries using the modified *L. plantarum* WCFS1 systems [47,48].

Driven by the challenge of GLP-1 delivery in combination with the possibility of flexible modification, and the advantage of targeted colonization of WCFS1, we explored a novel modified GLP-1 (mGLP-1) expression and oral delivery system. Combining *L. plantarum* WCFS1 with the modularly modified pSIP-system, we compared mGLP-1 secretion mediated by two endogenous signal peptides and selected one for subsequent morphological characterization and in vivo experiments in db/db mice to explore its biological activity.

## 2. Materials and Methods

### 2.1. Bacterial Strains, Plasmids, and Growth Conditions

The bacterial strains and plasmids used in this study were listed in Table 1. *Escherichia coli* (*E. coli*) DH5α was grown at 37 °C in LB medium with shaking at 220 rpm. *L. plantarum* WCFS1 was aerobically cultured statically at 37 °C in MRS medium. Solid media were prepared by adding agar (15 g/L) to the corresponding broth. Antibiotics were used at the final concentrations: 700 μg/mL erythromycin for *E. coli* and 5 μg/mL erythromycin for *L. plantarum*.

### 2.2. DNA Manipulation and Transformation

The codon-optimized m*glp-1* gene sequence with SP and His-tag was synthesized by GenScript Co., Ltd. (Nanjing, China). A plasmid extraction mini kit (TOROIVD, Shanghai, China) was used to isolate plasmid DNA from *E. coli* DH5α. The synthetic sequence, which contained an upstream *Ban*II and a downstream *PaeR7*I restriction site, was cleaved and ligated into the empty plasmid pSIP403 using these two restriction enzymes (New England Biolabs, Ipswich, MA, USA). After construction and confirmation by DNA sequencing and PCR, the pSIP403-p11-0600-mGLP-1, pSIP403-p11-0373-mGLP-1, and empty control pSIP403 plasmids were transformed into competent *L. plantarum* WCFS1 cells by electroporation.

### 2.3. Expression of Secreted mGLP-1

Overnight cultures of transformed *L. plantarum* WCFS1 were diluted 1:100 into fresh MRS containing 5 μg/mL of erythromycin, twice. An amount of 1% (*v*/*v*) of the above transformed *L. plantarum* WCFS1 was transferred into 10 mL fresh MRS medium containing 5 μg/mL of erythromycin and incubated statically at 37 °C for 12 h. Then the bacteria and supernatant were separated by centrifugation. The supernatant was precipitated with trichloroacetic acid (10% *w*/*v*) for 1 h at 4 °C, and the mixture was then centrifuged at 12,000 rpm for 10 min. The precipitate was washed three times with ice-cold acetone and finally dissolved in 100 μL PBS.

The concentrated culture supernatant was mixed with 5× SDS loading buffer and subjected to western blot analysis in a 16.5% Tricine-SDS-PAGE gel. Proteins were then semi-dry-electrotransferred onto a PVDF membrane, and binding was performed sequentially using the mouse 6× His antibody (Abcam, Cambridge, UK) and the peroxidase-conjugated rabbit-anti-mouse IgG secondary antibody (Abcam, Cambridge, UK). After washing three times, the membrane was subjected to ECL chemiluminescent kit (ShareBio, Shanghai, China) and analyzed. As for the enzyme-linked immunosorbent assay (ELISA), 10 μL of culture supernatant was sampled every 3 h of GLP-1 secretion and quantified by His-tag ELISA kit (Sangon Biotech, Shanghai, China) according to manufacturer’s instructions.

### 2.4. Scanning Electron Microscopy (SEM)

*L. plantarum* WCFS1 and transformed *L. plantarum* WCFS1 were grown in MRS medium for 24 h and subsequently fixed overnight using 4% paraformaldehyde. The bacteria collected were washed with various concentrations of ethanol solution and coated with platinum for 90 s. The morphological evaluation was then carried out with SEM using the HITACHI S-4800 (HITACHI, Tokyo, Japan) with an acceleration of 5 kV.

### 2.5. Animals and Experimental Design

Male C57BL/KsJ-db/db mice were supplied by Gempharmatech Co., Ltd. (Nanjing, China) and housed with access to water and standard laboratory chow in an air-conditioned room (22 ± 2 °C) with a 12 h light: 12 h darkness cycle. All animal experiments were conducted following relevant guidelines and regulations. Mice were kept for a one-week acclimation period and were 8-weeks-old at the start of the experiment. Mice were allocated into three groups of five animals with matching average body weights and blood glucose levels: (a) db/db mice treated with 200 uL W-pSIP403 (10^10^ CFU/mL) per day, (b) db/db mice treated with 200 uL W-0373-mGLP-1 (10^10^ CFU/mL) per day, and (c) control db/db mice treated with equal volume PBS per day. The oral dose of transformed probiotics was as described previously [25]. Non-fasting blood glucose and water intake were monitored at two-day intervals.

### 2.6. OGTT and Insulin Sensitivity Tests

The oral glucose tolerance test (OGTT) was performed according to Omori et al. with minor modifications [52]. On day 7 and 14 of the experiment, db/db mice were fasted overnight and given 1.5 g/kg body weight glucose via tube feeding of a glucose aqueous solution. Blood glucose concentrations were registered in blood samples collected from the tail vein before the tolerance test, and at 15, 30, 60, 90, 120 and 180 min after glucose administration. The area under the curves (AUCs) were then calculated.

Insulin sensitivity was assessed by determination of both the insulin tolerance test (ITT) and quantitative insulin sensitivity check index (QUICKI) [53]. For ITT, 4-h-fasted mice were intraperitoneally injected with 1 IU/kg body weight insulin. Glucose concentration in blood sampled from the tail vein was then detected before the test and at 30, 60, 90 and 120 min after insulin injection.

### 2.7. Plasma Biochemical Parameter Measurements

Blood samples were prepared via vein into a heparinized microcentrifuge tube and centrifuged for 10 min at 3000 rpm at the end of the experiment. Biochemical analysis of plasma lipid levels including high-density lipoprotein (HDL), low-density lipoprotein (LDL), triglyceride (TG) and cholesterol (CHO) levels were determined using the BS-360S automatic biochemical analyzer (MinDray, Shenzhen, China).

### 2.8. Histopathological Examination and Bacterial Counts

After 14-day administration, db/db mice were dissected. Tissues for histological analysis in each group were fixed with 4% paraformaldehyde, dehydrated in an alcohol-xylene system, and embedded in paraffin. Slices of pancreatic tissue at 5-μm thickness were subjected to hematoxylin and eosin (H&E) staining and immunohistochemical (IHC) analysis. The antibodies to glucagon and insulin were used for IHC analysis. Renal tissue sections (5 µm) were subjected to H&E and Masson staining analysis. The samples were all labeled with numbers. The average optical density (AOD) was calculated using Fiji Image J 1.49 (National Institute of Health, Bethesda, MD, USA).

The obtained colorectal tissues were rinsed with pre-cooled PBS before weighing. The tissues were then homogenized with 1 mL PBS and diluted according to the concentration gradient for erythromycin plate plating and subsequent CFU determination.

### 2.9. Data Analysis

Statistical analysis of the experimental data was performed using the GraphPad Prism 9.3.0 software (GraphPad Software Inc., San Diego, CA, USA) and SPSS for Windows 27.0 (IBM Corp., Armonk, NY, USA). For comparative analyses of normally distributed variables, significant differences between parameters such as plasma lipid levels and bacteria counts were calculated by the independent Student’s *t*-test or the one-way ANOVA. Differences between two groups of continuous variables such as the optical density (OD) value were evaluated using the independent Student’s t-test at each time point. Differences between three or more sets of continuous variables such as the non-fasting blood glucose, OGTT and ITT of mice were evaluated using the one-way ANOVA followed by Tukey’s multiple comparison post hoc test for the comparison at each time point. For more than two non-normally distributed data sets, the Kruskal-Wallis test was carried out. For data with non-homogeneity of variance such as QUICKI, Welch’s ANOVA test followed by Dunn’s multiple comparisons post hoc test was performed. The presented data are means ± standard deviation (SD). In all statistical analyses, a *p* value of <0.05 was considered to indicate a statistically significant difference.

## 3. Results

### 3.1. Construction and Expression of W-0373-mGLP-1

**Figure 1:**
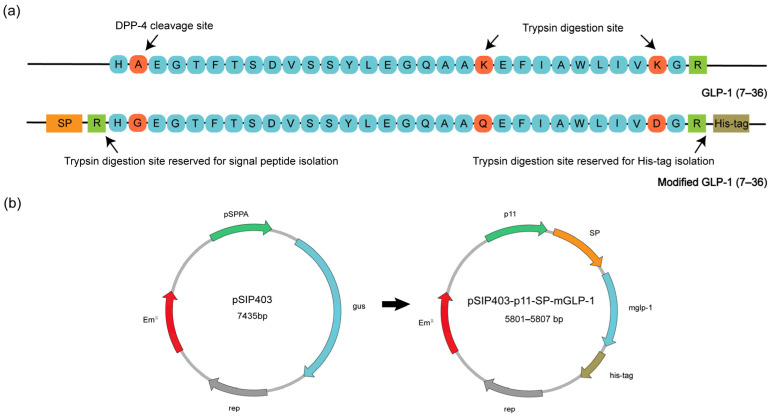
Schematic diagram of the modification of modified GLP-1 (mGLP-1) and plasmids. (**a**) Amino acid sequence comparison of native GLP-1 (7–36) and mGLP-1; (**b**) The schematic representation of the pSIP403 and constructed plasmid pSIP403-p11-SP-mGLP-1.

The DNA sequence of the mature active form of human GLP-1(7–36) was used as a template in this study, in which Ala8 was mutated to Gly8 to prevent DPP-4 recognition and cleavage. Lys26 and 34 were also mutated to Gln26 and Asp34 to inhibit trypsin digestion (Figure 1a) [54]. Promoter spacers for mGLP-1 expression were optimized as described before [55,56]. Signal peptides Lp_0600 and Lp_0373 were added in front of the modified *glp-1* gene for secretion, and arginine, the functional site of trypsin, was used as the dividing point. The arginine at the C-terminal of the m*glp-1* gene was retained and subsequently a His-tag was added to ensure the structural integrity of the released mGLP-1 peptide in vivo. After optimizing the codon sequence for expression in *L. plantarum* WCFS1 by the codon adaptation tool (JCAT, http://www.jcat.de/Start.jsp, accessed on 15 February 2022), the plasmids pSIP403, pSIP403-p11-0600-mGLP-1 and pSIP403-p11-0373-mGLP-1 were then electroporated into *L. plantarum* WCFS1 to produce the engineered strains for subsequent exploration (Figure 1b).

**Figure 2:**
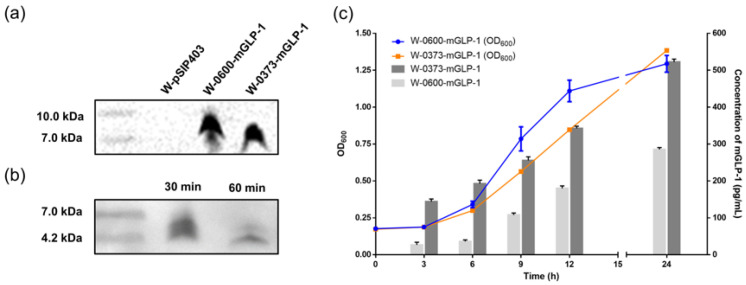
Western blotting and enzyme-linked immunosorbent assay (ELISA) analysis. Anti-His-tag antibody was used to detect mGLP-1 in supernatant. (**a**) The culture supernatant concentrated 50-fold of W-pSIP403 and W-SP-mGLP-1; (**b**) The supernatant of W-0373-mGLP-1 treated with 1 mL trypsin for 30 and 60 min; (**c**) The growth curves and ELISA of transformed strains at different time points. All data are represented as mean ± standard deviation (SD). OD: optical density.

The supernatants of the transformed *L. plantarum* WCFS1 were separated by centrifugation and collected after 6 h of inoculation as described above. The concentrated culture supernatant was subjected to Tricine-SDS-PAGE analysis via the C-terminal His-tag of mGLP-1. Bands of ≈7–10 kDa (expected: 9.3 and 9.1 kDa) were detected in the supernatant of the W-0373-mGLP-1, while no signal was detected in the control W-pSIP403 (Figure 2a). We confirmed that mGLP-1 expression was not detected in W-pSIP403 and that p11 and SP were required for the constitutive secretory expression of mGLP-1. We observed that the supernatant bands were around 9 kDa, indicating that the linked SP was not cleaved during secretion into the supernatant due to the acidic environment created by LAB as discussed before [57]. To ensure the integrity of the mGLP-1 for its in vivo biological activity, it is necessary to exclude the interference of SP with the His-tag. We treated the supernatant with trypsin and found that bands of ≈4–7 kDa were detected and attenuated, and no bands of other sizes appeared. This demonstrated the potential of designed arginine to release intact mGLP-1 by tryptic digestion (Figure 2b). Figure 2c showed growth curves and secretion of the transformed and control strains. W-0373-mGLP-1, which exhibited approximately 1.1-fold growth and 1.8-fold secretion potential at 24 h compared with W-0600-mGLP-1, was selected for subsequent in vivo experiments. Altogether, an oral delivery system of mGLP-1 by WCFS1 was constructed and screened preliminarily.

### 3.2. Scanning Electron Microscopy

**Figure 3:**
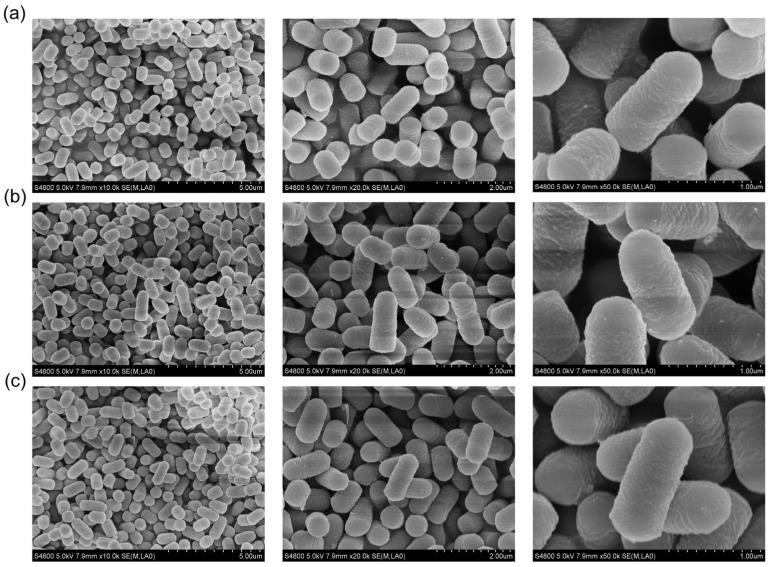
Bacterial morphology images of *Lactobacillus plantarum* (*L. plantarum*) WCFS1 and transformed *L. plantarum* WCFS1 samples using field emission scanning electron microscopy (SEM) at different magnifications. (**a**) *L. plantarum* WCFS1, (**b**) W-pSIP403 and (**c**) W-0373-mGLP-1.

Figure 3 presents the electron micrographs of *L. plantarum* WCFS1 and transformed *L. plantarum* WCFS1, respectively. The SEM photographs at a magnification of 50,000× showed intact rod-shaped bacteria structures with a rough, slightly wrinkled surface of all three groups. There were no obvious differences in surface, shape, or size between the transformed groups and the native strains.

### 3.3. Effects of a Single Dose of W-0373-mGLP-1 and Different Doses of Semaglutide on Blood Glucose

**Figure 4:**
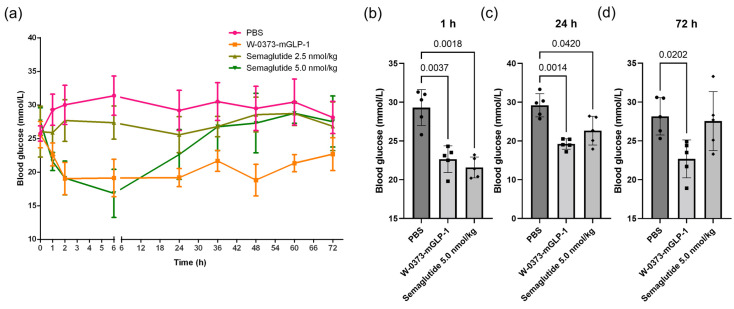
Effects of single dose administration of W-0373-mGLP-1 on non-fasting blood glucose in db/db mice. (**a**) Non-fasting blood glucose within 72 h following a single dose and at corresponding (**b**) 1, (**c**) 24, and (**d**) 72 h. All data are represented as mean ± SD.

Given that diabetes is often characterized by abnormal blood glucose, we investigated the effects of a single oral administration of the transformed probiotic and an intraperitoneal injection of positive control semaglutide on non-fasting blood glucose in db/db mice (Figure 4a). We found that non-fasting plasma glucose concentrations were reduced in all groups at 1 h compared with the control (Figure 4b). However, only the semaglutide (5 nmol/kg) and W-0373-mGLP-1 (10^10^ CFU/mL) were able to maintain lower blood glucose for approximately 24 h, after which, our transformed probiotics showed a more sustained glycemic maintenance for 72 h (Figure 4c,d). Taken these, a single oral administration of the engineered strain showed comparable improvement in blood glucose and a longer-lasting effect than the semaglutide injection.

### 3.4. Effects of 14-Day W-0373-mGLP-1 Administration on Blood Glucose, Random Water Intake, Fasting Blood Glucose, OGTT and ITT

**Figure 5:**
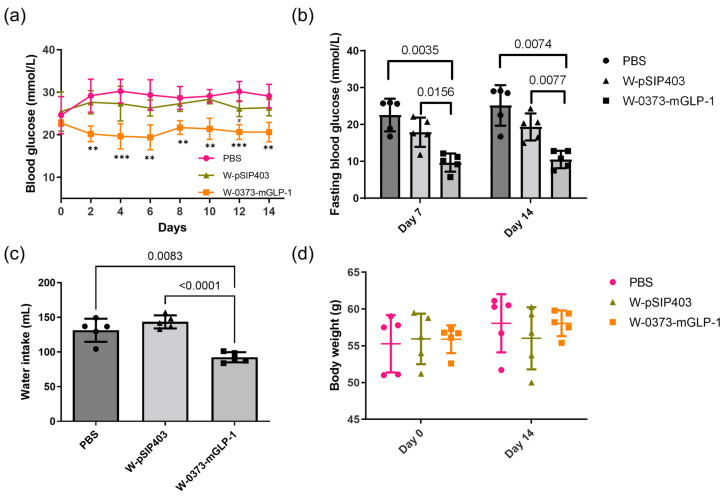
Effects of 14-day W-0373-mGLP-1 administration on (**a**) non-fasting blood glucose during administration, (**b**) 7- and 14-day fasting blood glucose, (**c**) random water intake per cage of different groups during administration in db/db mice, and (**d**) body weight. All data are represented as mean ± SD. ** *p* < 0.01, *** *p* < 0.001 compared with PBS group.

The antidiabetic effect was evaluated in the db/db mice by oral administration of W-0373-mGLP-1 or negative control W-pSIP403. Non-fasting blood glucose levels were measured every two days before administration during the 14-day period. The transformed strain produced a significant (*p* < 0.001–*p* < 0.01) decrease in blood glucose concentration since the second day of administration, whereas no significant differences were shown in the negative control groups throughout the treatment period except for day 12 compared with the control groups (Figure 5a). In addition, the fasting blood glucose was monitored on days 7 and 14 based on the certain contributions of its increase to total hyperglycemia [58]. The fasting blood glucose of control mice was higher than 22 mmol/L during the experiments, as shown in Figure 5b. While the fasting blood glucose of W-0373-mGLP-1 treatment group was about 10 mmol/L on days 7 and 14, which was significantly lower than that of the control group (*p* = 0.0035 and 0.0074, respectively). Similarly, water consumption on six randomly selected days of the administration group was lower than that in the PBS group and the negative control group with a statistical difference (*p* = 0.0083 and *p* < 0.0001), while no differences were found between controls and negative controls (Figure 5c). There was no change in body weight (Figure 5d), which may be related to the unchanged lifestyle [59].

**Figure 6:**
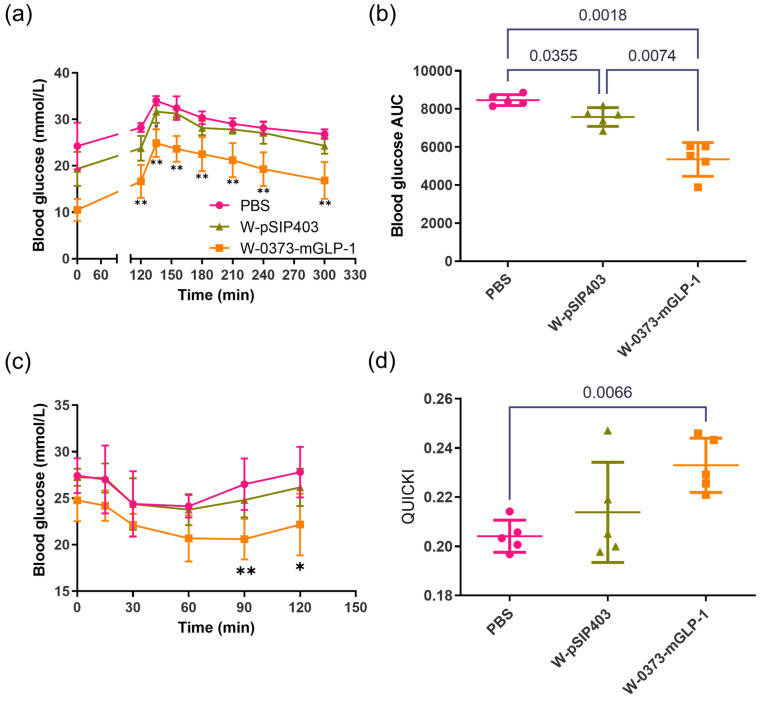
Effects of 14-day W-0373-mGLP-1 administration on (**a**) oral glucose tolerance test (OGTT) and (**b**) corresponding integrated glucose concentrations (AUCs), (**c**) insulin tolerance test (ITT) and (**d**) quantitative insulin sensitivity check index (QUICKI). All data are represented as mean ± SD. * *p* < 0.05, ** *p* < 0.01 compared with PBS group.

Following an intraperitoneal glucose injection, the blood glucose concentration and corresponding AUCs were measured until 180 min (Figure 6a,b). The blood glucose of db/db mice receiving W-0373-mGLP-1 treatment was significantly lower at all time points compared with db/db controls (*p* < 0.01). The corresponding area under the glucose curve was also significantly reduced (*p* = 0.0018). Although there was no significant difference in the OGTT curve between the PBS and W-pSIP403 groups, we were surprised to find that the W-pSIP403 strain also had a certain amelioration effect on the glucose tolerance in AUCs (*p* = 0.0355). In order to evaluate improvements in insulin sensitivity, exogenous insulin was administered intraperitoneally. The rates of glucose clearance from circulation were significantly better in W-0373-mGLP-1 treated groups at 90 min (*p* < 0.01) and at 120 min (*p* < 0.05) compared with controls (Figure 6c). Similarly, the QUICKI values, one of the indicators of insulin sensitivity, were significantly higher in the treatment group (*p* = 0.0066) (Figure 6d). Collectively, these data demonstrated that our transformed strains had the capability to alleviate some diabetic symptoms and improve insulin sensitivity in T2DM mice.

### 3.5. Effects of 14-Day W-0373-mGLP-1 Administration on Plasma Lipid Levels

**Figure 7:**
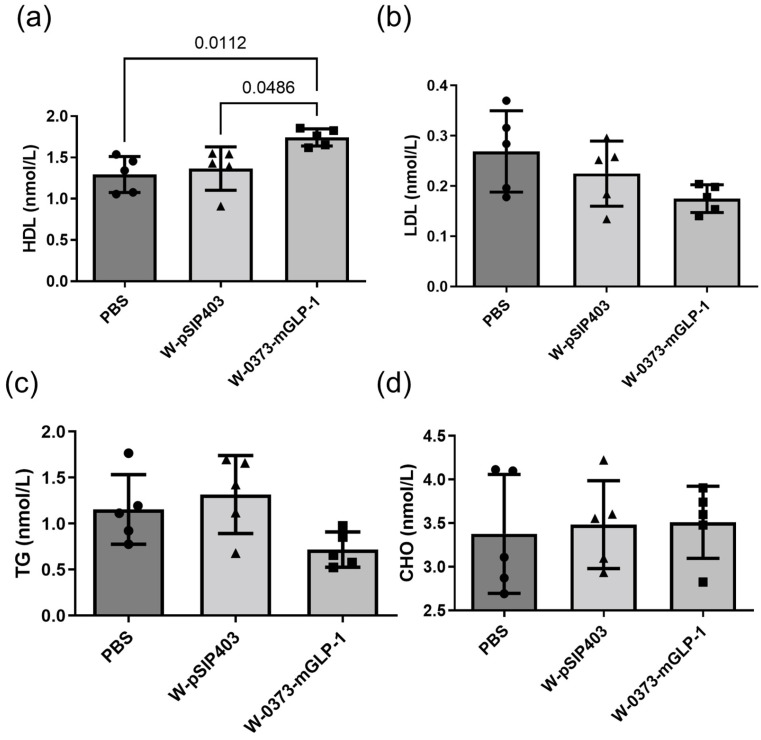
Effects of 14-day W-0373-mGLP-1 administration on plasma (**a**) high-density lipoprotein (HDL), (**b**) low-density lipoprotein (LDL), (**c**) triglyceride (TG), and (**d**) cholesterol (CHO) concentrations in db/db mice. All data are represented as mean ± SD.

T2DM is associated with a characteristic pattern of dyslipidemia, namely low HDL, elevated LDL, and TG levels [60]. To further evaluate the metabolic effects of administration, we measured various biochemical parameters in db/db mice. The plasma HDLs performed a slight but significant increase in mice treated with W-0373-mGLP-1 compared with controls (*p* = 0.0112) and negative controls (*p* = 0.0486) (Figure 7a). Similarly, the treatment lowered the LDL and TG levels compared with db/db controls although no statistical difference was produced (Figure 7b,c). The plasma CHO concentrations were not significantly altered in our treatment (Figure 7d). In summary, the administration of transformed probiotics had no significant effects on LDLs, TGs, and CHOs whereas circulating HDL concentrations were significantly increased. 

### 3.6. Effects of W-0373-mGLP-1 Administration on Pancreas

**Figure 8:**
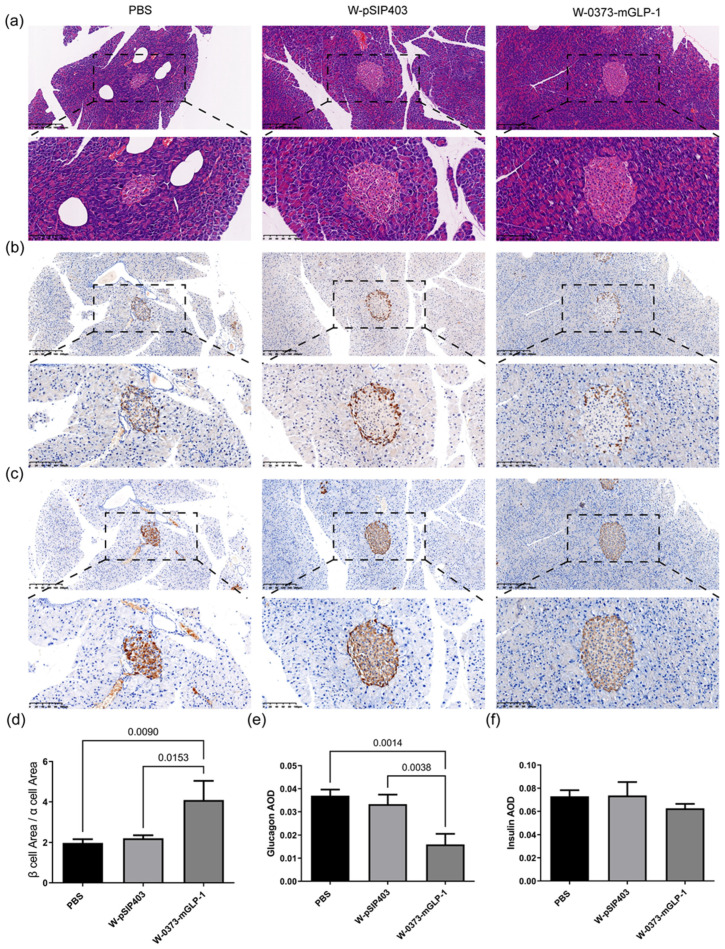
Effects of 14-day W-0373-mGLP-1 administration on pancreatic histomorphology, glucagon and insulin. Scale bar 20 μm (20× magnification). (**a**,**d**) Hematoxylin and eosin (H&E) staining of pancreas in db/db mice and corresponding ratio of β cells to α cells; (**b**,**e**) Immunohistochemistry (IHC) staining and quantitative average optical density (AOD) analysis of glucagon in pancreas; (**c**,**f**) IHC staining and quantitative AOD analysis of insulin in pancreas. All data are represented as mean ± SD.

The pancreatic cells dysfunction combined with insulin resistance are closely related to glucose dysmetabolism and the progression of T2DM [61]. Thus, the pancreatic histomorphology was investigated by H&E staining. The islets in the PBS group and W-pSIP403 group showed tissue atrophy resulting in a decrease in β cell area, which was alleviated by administration of the transformed strain (*p* = 0.0090 and 0.0153, respectively) (Figure 8a,d). Morphologically, the islets in the treatment group were elliptic, with clear boundaries and fewer empty areas than those in control group. In addition, glucagon secretion was enhanced in controls, exacerbating the diminishment of insulin release and hyperglycemia as shown in Figure 8b,e [62]. Treatment with W-0373-mGLP-1 significantly lowered the secretion of glucagon compared to both controls and negative controls (*p* = 0.0014 and 0.0038, respectively). Although administration of W-0373-mGLP-1 increased the number of insulin-secreting pancreatic β cells in islets, there was no significant difference in insulin secretion levels (Figure 8c,f). These findings indicated that administration of W-0373-mGLP-1 inhibited glucagon secretion and increased β-cell proportion.

### 3.7. Effects of 14-Day W-0373-mGLP-1 Administration on Kidney

**Figure 9:**
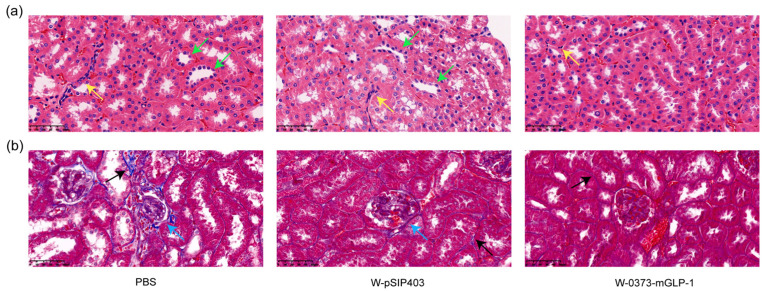
The protection effects of 14-day W-0373-mGLP-1 administration on kidney. Scale bar 10 μm (40× magnification). (**a**) H&E staining of kidney in db/db mice. Yellow arrow: inflammatory cells infiltration; green arrow: thickening of the renal tubules basement membrane; (**b**) Masson staining of kidney. Blue arrow: vacuoloid changes in tubular epithelial cells; black arrow: interstitial fibrosis.

Given that diabetic kidney disease (DKD) occurs in about half of all patients with T2DM and is the most common cause of death [63], we carried out renal pathological analysis. Representative images of the H&E staining showed significant inflammatory cells infiltration in the renal interstitium (yellow arrows) and irregular thickening of the renal tubule basement membranes in the control group. In contrast, there was only a slight infiltration of inflammatory cells in the treatment group (Figure 9a). Moreover, Masson staining of the kidney was conducted and the blue area represented the degree of renal fibrosis. After treatment with W-0373-mGLP-1, the blue area in the glomeruli and renal interstitium decreased compared with PBS group (black arrow). Glomerular cavity dilatation and glomerular atrophy with segmental adhesion of the balloon wall were observed in the control group, which were also relieved in the treatment group (blue arrow) (Figure 9b).

### 3.8. Colonization of L. plantarum in the Colorectum

**Figure 10:**
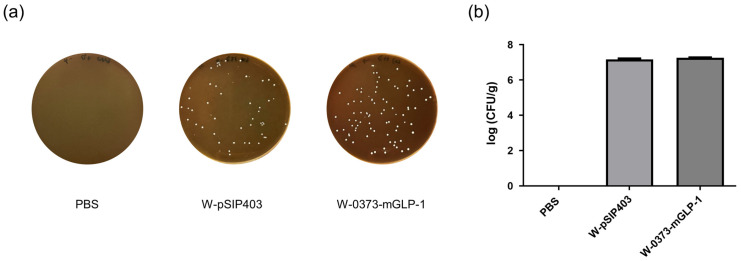
The density of the vehicle probiotics in the colorectum of treated db/db mice. (**a**) Plate-grown colonies of W-pSIP403 and W-0373-mGLP-1; (**b**) CFU of the vehicle probiotics in the colorectum. Each bar represents the average of the log-transformed count of colonies ± SD.

Given that the highest densities of *L. plantarum* were recorded in the cecum and colon [45], we chose the colorectum, where L-cells are located, to analyze its colonization. Figure 10a,b showed statistics of colonization in different groups after 14 days of continuous feeding. No statistically significant differences in counts was observed because of the sane amount of bacteria administered, which also proved that *L. plantarum* WCFS1 had a certain colorectal colonization ability and was not affected by our modification.

## 4. Discussion

In this study, we proposed a novel oral delivery system of the mGLP-1 by probiotics *L. plantarum* WCFS1. The model strain, *L. plantarum* WCFS1, was chosen due to the high colonization ability and survival capacity in the human gastrointestinal tract [51], which was also demonstrated in our results. In addition, *L. plantarum* WCFS1 contributes to extensive beneficial functions for human health such as immune modulatory capacities [51], blood lipids regulation [25], protective effects on the epithelial barrier [64], and intestinal environment improvement [65]. We aimed to achieve the oral delivery of mGLP-1 by a probiotics vehicle with extensive probiotic effects and colonization ability while improving the patient compliance of administration.

To improve the short circulation time of the native GLP-1 in vivo, we first constructed an mGLP-1 resistant to trypsin and DPP-4 enzymatic degradation based on the predominant active form of GLP-1 (7–36) in the intestine. Considering the importance of the free N-terminal of mGLP-1 [66], an arginine residue was designed after SP to avoid possible incomplete cleavage of SP. In addition, arginine at position 36 was retained to ensure the release of intact mGLP-1 in response to gastrointestinal digestive enzymes. The bioactivity of the corresponding peptides has been demonstrated previously [54]. The arginine function was also verified in vitro, and the absence of bands of other sizes as well as the weakening of the target band proved that trypsin could ensure the above cleavage.

To better mimic GLP-1 release from colonic L-cells in vivo, *L. plantarum* WCFS1 with colorectal colonization ability was used as the probiotic delivery vehicle for mGLP-1 [46]. The expression cassette used consisted entirely of endogenous accessories and the target gene was codon-optimized. Since different combinations of SP and protein might lead to discrepant secretion abilities, we selected two outstanding endogenous Lp_0373 or Lp_0600 as the guider for secretion [42,43]. We analyzed mGLP-1 secretion by ELSIA and found that the secretion of W-0373-mGLP-1 was approximately 1.8-fold more than W-0600-mGLP-1 at 24 h with the same starting strain amount. There was no obvious morphological change in the appearance of the transformed strains in the subsequent morphological characterization. Thus, the W-0373-mGLP-1 with higher secretion was finally selected for further exploration.

Furthermore, this work also demonstrated the long-term glycemic management advantage after a single oral administration. We compared the effects of the transformed strain on random blood glucose with that of the positive drug semaglutide of preclinical doses within 72 h after a single dose, and found that our transformed strain provided comparable and more durable effects [67]. Although the glycemic control of our mGLP-1 oral delivery system was attenuated at 72 h, the positive effects demonstrated the corresponding capacity as a long-acting GLP-1 receptor agonist for up to 3 days [68]. The continuous maintenance of blood glucose could be achieved by shortening the administration cycle.

To investigate the anti-diabetic effects of long-term administration, the changes in related indexes were observed during the 14-day daily administration period. In db/db mice, the progressive increase in non-fasting blood glucose concentrations was delayed significantly by treatment with the W-0373-mGLP-1 strain without impacting body weight. The impaired fasting glucose (IFG) of T2DM was also stably maintained at a lower level in T2DM compared with PBS-treated db/db mice. The additional water intake and resulted polyuria, which proved to be associated with glycemic regulation [69], was observed in controls and was alleviated in the treated group. In addition, the impaired glucose tolerance (IGT) was shown in db/db mice treated with PBS as a response to an intraperitoneal glucose load, while the response was improved significantly as was found in mice treated with the transformed strain. The treatment also had positive effects on the alteration of plasma lipid levels, especially HDL levels, reducing the linked cardiovascular risk [70]. These findings indicated that administration of W-0373-mGLP-1 protected the mice from hyperglycemia and other symptoms by inhibiting glucagon secretion and β-cell apoptosis as well as improving insulin sensitivity, which were consistent with the known effects of GLP-1 [7,8].

In T2DM, prolonged hyperglycaemia leads to the development of diabetic complications [71,72]. GLP-1 receptor agonists have been proven to be associated with significant beneficial cardiovascular effects, improvement in non-alcoholic fatty liver disease, and diabetic nephropathy [68,73,74]. Consistent with previous studies [74,75], the treatment of W-0373-mGLP-1 strain attenuated renal fibrosis and tissue injury compared with controls. In addition, reduced inflammatory cell infiltration was found in both W-pSIP403 and W-0373-mGLP-1 groups, which might also be related to the ameliorating effect of *L. plantarum* WCFS1 on inflammation [76], suggesting the potential of the probiotic vehicle application in T2DM.

Bio-therapeutic applications of probiotics have progressed rapidly in the past decade, with several clinical studies approved [35,77], paving the way for other probiotics-delivered applications. The increased attention is closely related to the expression flexibility of this novel delivery system and its beneficial effects on the target diseases [25,78,79]. With the modification of different strains of LAB, several treatment options for DM provided by probiotics are also gradually emerging [54,57,79,80]. Compared with other oral mucosal targeted delivery systems of the GLP-1 receptor agonist, the LAB delivery vehicle exhibits sustained expression and release ability, recognized biosafety [20], and extensive probiotic effects (Table 2) [25,51]. It is worth mentioning that the LAB delivery system, like other mucosal-targeted delivery systems, showed inspiring intestinal transport of proteins, which may be related to the slight opening of cellular tight junctions and local high-concentration gradients of target proteins due to bacterial adhesion to intestinal cells [57,80]. The limited benefits observed in previous studies may be due to the structural obstruction at the N-terminal of GLP-1 and the differential colonization abilities of the LAB vehicles.

To summarize, our construction might include several advantages as follows. First, several appropriate modifications were applied to GLP-1 (7–36) to prolong its circulation time based on known biological activities of corresponding peptides and to ensure the release of intact mGLP-1 in vivo. Second, the bio-safe probiotic *L. plantarum* WCFS1, with in situ colonization ability and extensive probiotic effects, was used as the vehicle to ensure the oral delivery of mGLP-1 to the intestine. Furthermore, constitutive expression was achieved with the flexible combination of endogenous promoter and signal peptides, reversing the inefficient expression led by the incompatible heterologous elements. Nevertheless, for the LAB vehicle with the highest densities of colonization in the colorectum, special populations, such as T2DM patients after colorectal resection or those requiring anti-infective therapy, need to combine other treatments to ensure effective glycemic control.

## 5. Conclusions

Taken together, we proposed a novel oral delivery and expression system of mGLP-1 as a long-acting GLP-1 receptor agonist. The probiotic delivery system of mGLP-1 was effective in alleviating diabetes symptoms and had the potential to relieve diabetic kidney disease in db/db mice. These changes were related to a decreased pancreatic glucagon, elevated pancreatic β-cell proportion, and increased insulin sensitivity. Additionally, the *L. plantarum* WCFS1 delivery vehicle, which has the ability to colonize the colon, also exhibited probiotic effects on OGTT and inflammation. The combination of therapeutic molecules with probiotics with related probiotic effects also provides new options for the treatment of other diseases. Further studies on the pharmacokinetics of the mGLP-1 release and the combination of more efficient expression elements in the probiotic delivery systems are pending.

## Figures and Tables

**Table 1:** Plasmids and bacterial strains used in this study.

Plasmids/Strains	Description	Source
Plasmids		
pSIP403	Em^R^, containing the pSPPA promoter	[49]
pSIP403-p11-0600-mGLP-1	pSIP403 carrying promoter p11, signal peptide Lp_0600 and modified *glp-1* gene	This study
pSIP403-p11-0373-mGLP-1	pSIP403 carrying promoter p11, signal peptide Lp_0373 and modified *glp-1* gene	This study
Strains		
*Escherichia coli* (*E. coli*) DH5α	Host strain	[50]
*Lactobacillus plantarum (L. plantarum)* WCFS1	Host strain	[37,51]
W-pSIP403	*L. plantarum* WCFS1 containing empty plasmid pSIP403	This study
W-0600-mGLP-1	*L. plantarum* WCFS1 containing plasmid pSIP403-p11-0600-mGLP-1	This study
W-0373-mGLP-1	*L. plantarum* WCFS1 containing plasmid pSIP403-p11-0373-mGLP-1	This study

GLP-1: glucagon-like peptide-1, Em^R^: erythromycin resistance.

**Table 2:** A brief description of the oral mucosal delivery systems for GLP-1 receptor agonists.

Technique	Details of Formulation	Antidiabetic Peptides	Advantages	Reference
Permeation enhancer	sodium N-(8-[2-hydroxybenzoyl] amino) caprylate (SNAC)	Semaglutide	Promoting the transport of polypeptides across the gastrointestinal biofilm	[18]
Mucoadhesive polymer	Chitosan	Exendin-4	Mucosal adhesion properties, high stability against enzymatic degradation	[81]
Nanostructured lipid carrier	Solid lipid	GLP-1/Exendin-4	Activating the endogenous GLP-1 secretion, site-specific release	[82]
Nanoparticle	Chitosan—poly(lactide-co-glycolide) polymer	GLP-1	Protecting peptides from degradation and promoting the permeation of GLP-1 across the intestinal	[83]
Lactic acid bacteria (LAB) delivery system	LAB	GLP-1/Exendin-4	Functioning as a vehicle both for expression and delivery, bio-safety, colorectal colonization ability, intestinal transport capacity, treatment-associated probiotic effects	[57,80]

## Data Availability

All the relevant data are available from the corresponding authors upon reasonable request.
